# Concurrent Cardiac and Renal Anomalies in Waardenburg Syndrome Type 1: A Report of a Rare Case

**DOI:** 10.7759/cureus.63206

**Published:** 2024-06-26

**Authors:** Ameer Awashra, Ahmad Nouri, Thabet Zidan, Abdelrahman Sawalma, Fathi S Milhem, Dalia Marbo’

**Affiliations:** 1 Faculty of Medicine and Health Sciences, An-Najah National University, Nablus, PSE; 2 Critical Care Nursing, H-Clinic Hospital, Ramallah, PSE

**Keywords:** pax3 gene mutation, melanocyte disorders, cardiac anomalies, renal anomalies, waardenburg syndrome type 1

## Abstract

Waardenburg syndrome (WS) is an autosomal dominant genetic disorder characterized by the absence of melanocytes, leading to distinctive pigmentary abnormalities and sensorineural hearing loss. This case report describes extremely rare concurrent anomalies in a preterm male infant diagnosed with WS type 1.

The newborn, delivered prematurely at 35 weeks due to maternal complications, presented with multiple congenital anomalies and required immediate resuscitation. He exhibited hallmark features of WS, including a white forelock, dystopia canthorum, and bilateral sensorineural hearing loss. Genetic testing confirmed a PAX3 gene mutation. The infant experienced significant respiratory and feeding challenges, necessitating intensive care. Management included mechanical ventilation, surfactant therapy, phototherapy for hyperbilirubinemia, and broad-spectrum antibiotics for suspected sepsis. The cardiac assessment revealed multiple anomalies, such as a patent foramen ovale and left ventricular hypertrophy, while renal ultrasound identified multicystic dysplastic kidney and bilateral hydronephrosis. Multidisciplinary care facilitated the infant's stabilization, transition to oral feeding, and ongoing specialized care.

WS type 1 is associated with mutations in the PAX3 gene and presents with diverse clinical manifestations. Although renal and cardiac anomalies are uncommon in WS, their presence in this case underscores the complexity of the syndrome. Early intervention for hearing impairment and genetic counseling are critical for optimal outcomes. This report highlights the importance of a comprehensive and interdisciplinary approach to managing infants with WS, addressing both typical and atypical manifestations. It is worth noting that effective management of WS in neonates requires prompt identification and treatment of associated complications.

## Introduction

Waardenburg syndrome (WS) represents an autosomal dominant genetic disorder typified by the absence of melanocytes in the eyes, hair, and skin structures [1], leading to its classification as an auditory-pigmentary syndrome. Clinically, it is delineated by hallmark pigmentary disturbances of the hair, notably the presence of a white forelock and premature graying; ocular pigmentary discrepancies, including heterochromia irides and brilliant blue irises; in conjunction with congenital sensorineural hearing loss [2]. The global incidence of WS is estimated at two per 100,000 individuals [3].

We report a preterm male newborn with a predisposition to WS, who presented with multiple complications at birth. Despite early respiratory and feeding challenges, he stabilized with no neurological issues. Genetic testing confirmed WS and multisystem anomalies were managed effectively. The infant’s recovery was supported by a multidisciplinary team, ensuring a successful transition to regular oral feeding and ongoing specialized care.

## Case presentation

A newborn male with a strong genetic predisposition to WS was born prematurely at 35 weeks of gestation via urgent cesarean section due to complications, including meconium aspiration, gestational hypertension, and maternal diabetes mellitus. He weighed 3400 grams at birth. The delivery required immediate resuscitative measures as the infant was flaccid and cyanosed. Apgar scores were 2 and 6 at one and five minutes, respectively, and special management of an umbilical cord injury was necessary.

Upon admission to the neonatal intensive care unit (NICU), the infant exhibited symptoms consistent with transient tachypnea of the newborn (TTN). The initial assessment revealed moderate respiratory distress characterized by mild subcostal and intercostal retractions, with an initial oxygen saturation of 88% on room air. Echocardiography showed no evidence of persistent pulmonary hypertension of the newborn (PPHN). Given the clinical picture, the infant was promptly admitted to the NICU for specialized care.

Treatment began with supplemental oxygen via nasal cannula, but due to worsening respiratory status, including increased work of breathing and intermittent desaturation, it was intensified to endotracheal intubation and mechanical ventilation. The highest ventilator settings recorded were a peak inspiratory pressure (PIP) of 20 cm H2O, positive end-expiratory pressure (PEEP) of 4 cm H2O, a fraction of inspired oxygen (FiO2) of 40%, and a rate of 30 breaths per minute.

**Figure 1:**
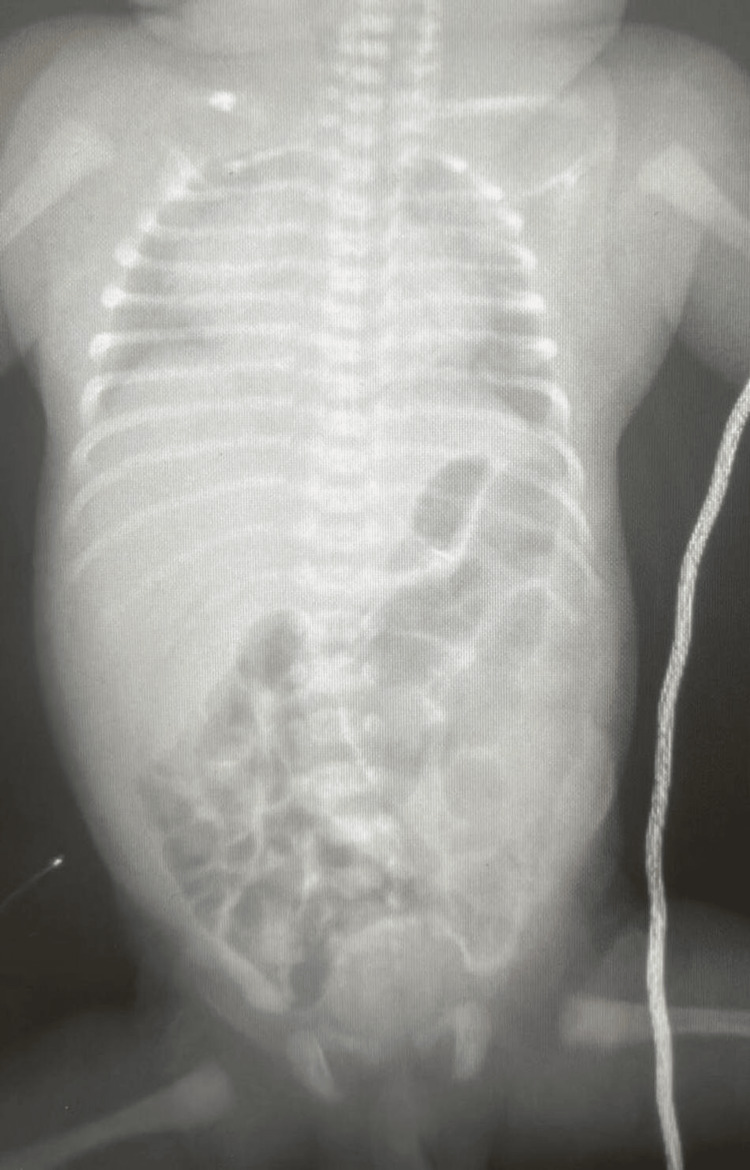
Admission chest X-ray revealed widespread pulmonary opacification and a modestly enlarged heart contour.

Initial radiographs showed diffuse pulmonary haziness and a slightly enlarged heart, consistent with TTN, but these abnormalities gradually resolved with continued supportive care (Figure 1).

**Figure 2:**
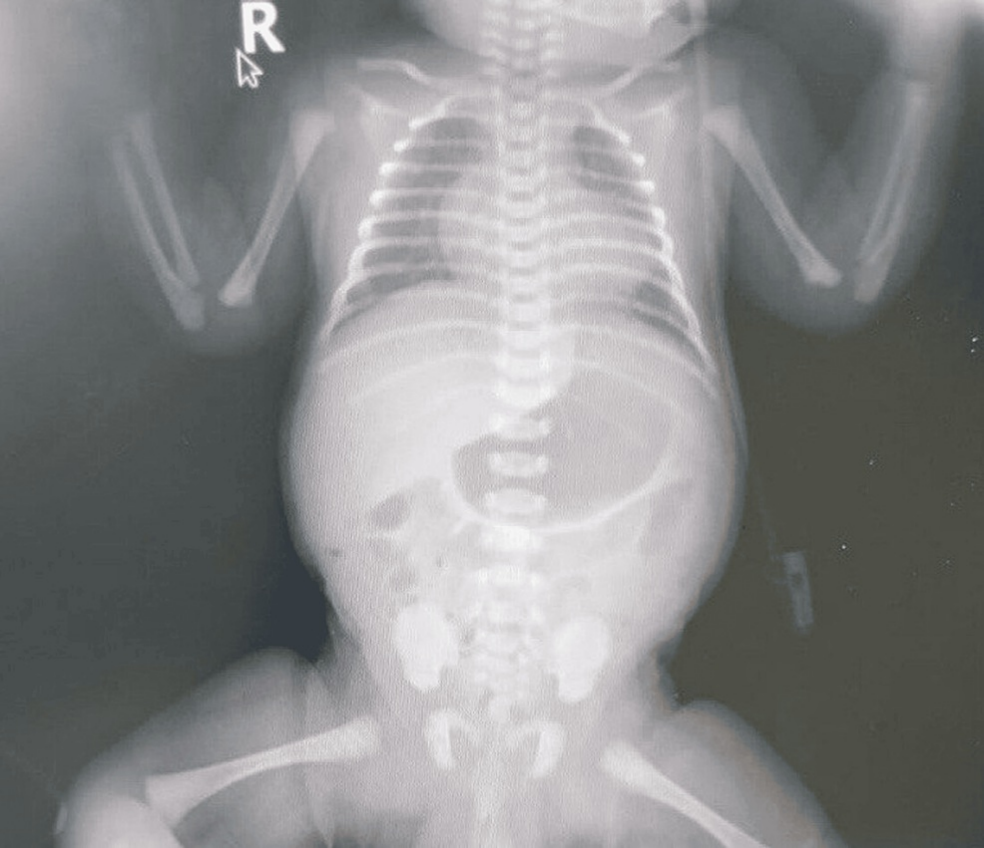
Chest X-ray on day five revealed a marked resolution of pulmonary haziness and a notable reduction in cardiac silhouette size, indicating improvement in the patient’s condition.

Despite standard ventilatory support, the infant continued to struggle with hypoxemia. This led to a brief period of high-frequency oscillatory (HFO) ventilation before a return to conventional methods. Alongside surfactant therapy, the medical team made frequent adjustments to the ventilatory settings, closely monitored blood gas levels (Table 1), and conducted serial chest X-rays (Figure 2).

**Table 1:** Comparative laboratory values on admission day, day 5, and discharge day from the pediatric unit. pCO2: partial pressure of carbon dioxide; pO2: partial pressure of oxygen; HCO3: bicarbonate.

Blood test	Admission day	Day 5	Discharge day	Normal range	Unit
Complete blood count (CBC)					
Hemoglobin (HGB)	16.6	14.8	11.5	9.5-14.5	g/dL
Hematocrit (HCT)	52.9	45.1	34.1	31-41	%
Red blood cells (RBC)	4.88	4.42	3.77	3.9-5.3	10^6^/uL
Mean cell hemoglobin concentration (MCHC)	31.4	32.82	33.7	31-35	g/dL
Mean cell hemoglobin (MCH)	34	33.48	3.5	27-31.2	pg
Mean cell volume (MCV)	108	102	90.5	80-97	fL
Platelets count (PLT)	327	243	525	150-450	10^3^/uL
White blood cells (WBC)	38.1	16.3	8.59	5.4-13.8	10^3^/uL
Neutrophils	11.3	5.43	2.23	1.7-7.7	10^3^/uL
Lymphocytes	24.1	5.64	4.32	0.7-4.8	10^3^/uL
Monocytes	1.64	1.5	1.28	0.0-0.9	10^3^/uL
General chemistry					
Sodium	138.7	142	138.1	135-145	mEq/L
Potassium	4.18	4.9	4.7	3.5-5.3	mEq/L
Arterial blood gases (room air)					
pH	7.224	7.4	7.413	7.35-7.45	-
pCO2	55.8	34.4	39.9	35-45	mmHg
pO2	46.1	55.8	67.3	83-108	mmHg
HCO3	22.5	21.5	24.9	22-26	mEq/L
Others					
C-reactive protein (CRP)	0.05	5.2	0.08	Less than <0.5	mg/dl
Bilirubin	5.7	12.7	4.8	2-6	mg/dl
Creatinine	1.3	0.9	0.72	0.40-1.00	mg/dl

Sepsis management involved treating elevated inflammatory markers with broad-spectrum antibiotics (Table 1), despite negative blood cultures. The antibiotics were initially started at admission to the NICU due to the generalized sickness level of the baby, even though the initial absolute neutrophil count (ANC) was not low and CRP was normal. This was a precautionary measure due to the severe clinical presentation, including generalized hypotonia and bulging open anterior fontanelle. On day five, with CRP being elevated, the sepsis management continued with broad-spectrum antibiotics, leading to the normalization of inflammatory markers and resolution of clinical signs of sepsis. Meningitis was ruled out via lumbar puncture.

On the third day after birth, the infant’s hyperbilirubinemia reached its highest level, with total serum bilirubin (TSB) levels peaking at approximately 10-12 mg/dL (171-205 µmol/L). Effective management with phototherapy was initiated, as this value exceeded the cut-off for a premature infant (35-37 weeks gestation). The total duration of phototherapy was six days. The maximum TSB value recorded was 14.5 mg/dL. This treatment resulted in a gradual reduction of bilirubin levels. To ensure the infant’s well-being, bilirubin levels continued to be carefully monitored following the phototherapy treatment to prevent any rebound hyperbilirubinemia. Apart from suspected sepsis, risk factors for hyperbilirubinemia included prematurity and potential genetic predispositions.

The cardiac assessment uncovered a spectrum of anomalies. Notably, there was a patent foramen ovale with a left-to-right shunt and left ventricular hypertrophy of the non-obstructive variety. Additionally, a moderate tricuspid valve regurgitation was measured at 60 mmHg, alongside a small muscular ventricular septal defect. A significant finding was a large patent ductus arteriosus (PDA), measuring 5 mm with a bidirectional shunt, which fortunately demonstrated considerable improvement over time. The shunt direction was predominantly left-to-right as it became more restrictive with a maximum gradient of approximately 20 mmHg. The left atrium (LA) ratio and superior mesenteric artery (SMA) Doppler were not specified, and no features indicated hemodynamic compromise. These cardiac abnormalities were managed with a conservative approach, showing no indications of heart failure. Despite requiring heightened respiratory support to HFO due to severe respiratory distress, the PDA was managed conservatively because of features suggestive of PPHN, where maintaining a PDA can be beneficial.

The abdominal ultrasound brought to light a series of renal abnormalities. Among these was a left-sided multicystic dysplastic kidney, accompanied by bilateral hydronephrosis. The left ureter appeared tortuous and dilated, extending all the way to the ureterovesical junction, which was associated with a grade 4 vesicoureteral reflux (VUR). On the other hand, the right ureter showed mild changes consistent with grade 2 VUR. These findings are corroborated by a voiding cystourethrogram. Additionally, there was a suggestion of a duplicated collecting system on the left side. Initial renal function tests indicated elevated creatinine levels, but these levels showed improvement over time (Table 1). As a preventive measure, prophylactic antibiotics were prescribed.

In the first few days, the infant’s nourishment was carefully managed without oral feeding, progressing cautiously to bottle feeding. Initially, the baby faced challenges due to a weak sucking reflex and the presence of thick saliva. To ensure proper nutrition, breast milk was administered through a feeding tube, with the quantity gradually increased over time. As the baby approached discharge, he had developed the ability to feed normally, demonstrated healthy weight gain, and showed excellent tolerance for feeding. Additional nutritional support and guidance for breastfeeding were provided, fostering the baby’s continued growth and development.

During the patient's hospitalization, multiple clinical signs raised suspicions of WS, as the patient exhibited several distinctive facial features. These included dystopia canthorum, telecanthus, a striking white forelock, and a notably broad nasal root and bridge. A thorough examination revealed no signs of organomegaly. The patient's male genitalia were within normal parameters, and no skeletal abnormalities were detected. In light of this presentation, a series of tests were recommended, including chromosomal analysis, genetic screening, and hearing evaluations.

Upon conducting an auditory brainstem response test, the infant was diagnosed with bilateral sensorineural hearing loss. The infant exhibited neither abnormal movements nor signs of seizures. Additional neurological examinations and brain ultrasound scans were conducted, all of which returned normal findings with no evidence of ventriculomegaly, intraventricular hemorrhage, or structural anomalies.

The diagnosis of WS in the patient’s father, coupled with the distinctive white forelock observed in four cousins, led to a comprehensive genetic investigation. The recurring appearance of this trait within the family pointed to a strong genetic predisposition, suggesting the likelihood of an inherited disorder. Detailed genetic testing yielded a heterozygous mutation in the PAX3 gene on chromosome 2q36 (Online Mendelian Inheritance in Man (OMIM): 606597). This particular genetic variation is characteristic of WS type 1, which aligns with the phenotypic patterns documented among the relatives.

Following the patient’s discharge, a scheduled follow-up plan was established, involving regular evaluations by both a urologist and a cardiologist. These consultations are intended to closely monitor the patient’s recuperation and to maintain the highest standard of care for their ongoing health.

## Discussion

WS represents a rare cluster of autosomal dominant genetic disorders that embryologically influence the dispersion of melanocytes, resulting in depigmentation across diverse anatomical sites due to the depletion of pigmentary cells in the hair, skin, eyes, and cochlear stria vascularis. Additionally, documented phenotypic presentations encompass a broad nasal root, deaf-mutism, depigmented forelock, aberrantly pigmented iris, hypertrichosed medial eyebrows, and lateral displacement of medial canthi with dystopia of lacrimal puncta [4].

Various types of gene mutations contribute to the onset of WS, including frameshifts, deletions, insertions, missense, and nonsense mutations. Four distinct types of WS have been delineated, with the initial two types being the most prevalent, while the third is exceptionally rare. Classification of these types is based on the specific mutated gene; notably, type 1 arises from a mutation in the PAX3 gene, situated within the 2q36.1 region [4-6].

Our patient was suspected to have WS type 1 due to the presence of characteristic features (the second, fourth, and fifth major criteria in addition to the third minor criterion) that align with the diagnostic criteria outlined in Table 2.

**Table 2:** Diagnostic criteria for Waardenburg syndrome type 1 with the probability of the presence of some of the characteristics. * Irides of entirely different colors in each eye. ** Presence of two different colors within the same iris, most commonly a combination of brown and blue segments. *** The W index, measured in millimeters (mm), is calculated using the following measurements: (a) inner canthal distance, (b) interpupillary distance, and (c) outer canthal distance. The calculations are performed as follows: X = (2a – [0.2119c + 3.909])/c; Y = (2a – [0.2479b + 3.909])/b; W = X + Y + a/b. An index value greater than 1.95 is indicative of dystopia canthorum. Modified from references [5,7].

Major criteria	
1	Congenital sensorineural hearing loss (47%-58%)
2	White forelock, hair hypopigmentation (43%-48%)
3	Pigmentation abnormality of the iris, and can include one or more of the following: (A) complete heterochromia iridum (15%-31%)*; (B) partial/segmental heterochromia**; (C) hypoplastic blue irides or brilliant blue irides (15%-18%)
		4	Dystopia canthorum, W index > 1.95***
		5	Affected first-degree relative
		Minor criteria	
1	Skin hypopigmentation (congenital leukoderma) (22%-36%)
2	Synophrys and/or medial eyebrow flare (63%-73%)
3	Broad/high nasal root, low-hanging columella (52%-100%)
4	Underdeveloped alae nasi
5	Premature gray hair (age < 30 years) (23%-38%)

The diagnosis of WS is confirmed if the patient satisfies either two major criteria or one major criterion along with two minor criteria, or upon detection of a heterozygous pathogenic variant in PAX3 [5,7]. Additionally, although rare, other reported manifestations of WS encompass cleft lip and/or palate, broad square jaw, low anterior hairline, spina bifida, limb defects, Hirschsprung disease, Sprengel anomaly, and abnormalities of vestibular function [7].

There is no definitive treatment for WS; however, management primarily aims to enhance the patient’s quality of life by addressing some of the abnormal manifestations. Early recognition and treatment of hearing abnormalities are crucial for normal mental development, with cochlear implantation being a definitive intervention. Protection from ultraviolet rays is important for the hypopigmented areas that are easily sun-damaged. Additionally, genetic counseling is a critical aspect to discuss with the family [4,5].

Children with WS have a normal life expectancy. However, neural-crest-derived tissue-related defects are the main causes of morbidity, which include psychiatric and mental disabilities, skeletal anomalies, ocular disorders, and hearing abnormalities [4].

Cardiac and renal manifestations are extremely unlikely to be associated with WS. To our knowledge, two cases of WS have been reported with cardiac anomalies, and only four cases in the literature have been found to have renal anomalies. Among these, two cases had multicystic dysplastic kidney and hydronephrosis, which are similar renal findings to our case. Other reported renal anomalies include duplicated ureteral collecting system and horseshoe kidneys [8-12].

A plausible theory regarding the potential association between WS and renal anomalies suggests that neural crest cells migrate to the embryonic kidney during urinary system development, where they form nephrogenic mesenchyme. This mesenchyme then triggers the differentiation and formation of the kidney [13]. While congenital kidney malformations can arise from aberrant neural crest cell migration, as demonstrated in prior studies, it remains to be thoroughly investigated whether renal abnormalities serve as symptoms of WS [10].

The management of renal issues in patients with WS varies depending on the specific anomalies present. For instance, a duplicated collecting system typically necessitates no treatment unless it is concomitant with another anomaly such as ureteropelvic junction obstruction or VUR [8,12]. Conversely, patients with a multicystic dysplastic kidney typically require nephrectomy, which is the treatment of choice for this condition [9]. The management of VUR spans from continuous antibiotic prophylaxis to various types of urological surgeries [14].

## Conclusions

Finally, we highlight the intricate management of a newborn diagnosed with WS type 1, emphasizing the necessity for a thorough and interdisciplinary approach to complex genetic conditions. The infant exhibited hallmark symptoms of WS, along with unanticipated renal and cardiac abnormalities, which called for meticulous neonatal care and genetic evaluation.

Prompt and effective treatment of critical conditions, including transient tachypnea and hyperbilirubinemia, as well as specialized management of renal and cardiac complications, highlighted the significance of prompt and ongoing care. Furthermore, this case accentuated the vital importance of addressing early hearing impairment and providing genetic counseling to the family, reinforcing the need for early detection and comprehensive care strategies.
